# On the Correlation Between Gastrointestinal Symptoms and Sites for Endoscopic Biopsies to Diagnose Graft-Versus-Host Disease

**DOI:** 10.14309/ctg.0000000000000950

**Published:** 2025-11-21

**Authors:** Carlos Figueredo, Melissa Fazzari, Lawrence J. Brandt

**Affiliations:** 1 Division of Gastroenterology and Hepatology, Montefiore Medical Center, Bronx, New York, USA;; 2 Department of Biostatistics, Albert Einstein College of Medicine, New York, New York, USA.

**Keywords:** cost-efficiency, endoscopy, GVHD, healthcare expenditure

## Abstract

**INTRODUCTION:**

Gastrointestinal graft-versus-host disease (GI-GVHD) is a serious complication of hematopoietic stem cell transplantation, with diagnosis reliant on results of endoscopic biopsy. Optimal endoscopic approaches based on symptoms remain unclear.

**METHODS:**

We conducted a retrospective cohort study of 75 adult hematopoietic stem cell transplantation recipients with GVHD and GI symptoms undergoing endoscopic biopsy at Montefiore Medical Center (2015–2023). We assessed correlations between presenting upper (UGI) or lower GI (LGI) symptoms and biopsy-proven GVHD. Statistical analyses included χ^2^ tests, phi coefficients, and logistic regression adjusting for demographic confounders. A subgroup analysis compared the diagnostic yield of flexible sigmoidoscopy vs full colonoscopy.

**RESULTS:**

Biopsy positivity strongly correlated with symptom location: 89.5% of patients with UGI symptoms had positive upper GI biopsies, and 100% with LGI symptoms had positive lower GI biopsies. χ^2^ tests showed significant associations between symptoms and biopsy positivity (*P* < 0.001), with phi coefficients indicating strong correlations (*r* = 0.79 UGI; *r* = 0.82 LGI). Logistic regression confirmed symptom location as an independent predictor of biopsy results. Among 37 patients undergoing full colonoscopy, rectosigmoid biopsies showed perfect correlation with a diagnosis of GVHD (*r* = 1.0, *P* < 0.001) when compared with other anatomic colon biopsy sites, suggesting flexible sigmoidoscopy is a cost-effective alternative for LGI symptoms.

**DISCUSSION:**

Symptom-guided endoscopic evaluation in GI-GVHD yields high diagnostic accuracy. Flexible sigmoidoscopy with targeted biopsies should be considered for patients with LGI symptoms because it may reduce procedural burden and health care costs without compromising diagnostic yield. These findings support symptom-directed, anatomically targeted approaches to improve patient care and resource utilization.

## Graphical abstract

**Figure:**
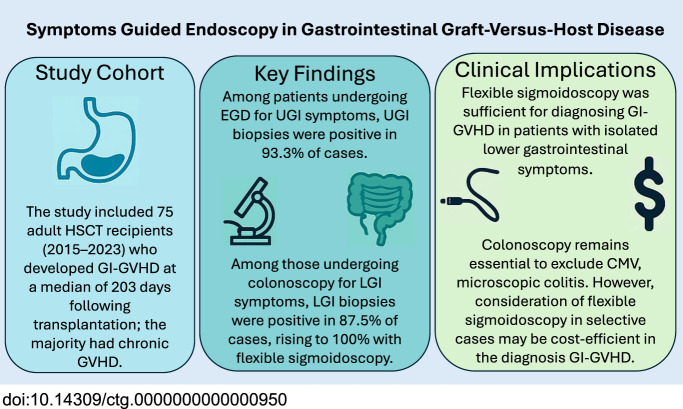

## INTRODUCTION

Graft-versus-host disease (GVHD) is a severe and potentially life-threatening complication after hematopoietic stem cell transplantation (HSCT), which affects approximately 30%–50% of patients receiving HLA-matched sibling transplants and up to 60%–90% of those undergoing mismatched transplants (1). The gastrointestinal (GI) tract is the second most commonly involved organ system in GVHD after skin and often leads to significant morbidity from symptoms such as nausea, vomiting, diarrhea, and bleeding. Prompt and accurate diagnosis of GI-GVHD is critical for initiating appropriate immunosuppressive therapy and improving patient outcomes.

Endoscopic biopsy remains the diagnostic gold standard for confirming GI involvement in GVHD. However, uncertainty persists regarding the optimal endoscopic approach and biopsy site selection based on presenting symptoms. Traditionally, the upper GI tract (stomach and duodenum) is biopsied in patients with nausea and vomiting, while the small intestine or colon is targeted in those with diarrhea. Despite these clinical guidelines, the sensitivity and diagnostic yield of biopsies from different GI sites remain controversial (2,3).

Several studies have addressed this issue but report conflicting results. For example, Snover et al demonstrated that among patients with diagnostic upper GI biopsies, only 59% had concomitant positive rectal biopsies (3). Conversely, other studies reported higher diagnostic yields from distal colon biopsies compared with upper GI sites (4,5). These discrepancies may be due to small sample sizes, heterogeneous patient populations, and limited ethnic diversity in earlier research.

Furthermore, the current literature lacks robust evidence on whether GI symptom presentation can reliably guide the choice of endoscopic procedure—an important consideration to avoid unnecessary invasive procedures, reduce patient discomfort, and decrease health care costs. The economic burden of excessive or redundant endoscopic evaluations is substantial, particularly in resource-limited health care settings.

Our study addresses these gaps by leveraging a large, ethnically and culturally diverse patient population from the Bronx, NY, to examine the relationship between presenting GI symptoms and diagnostic yields of targeted upper and lower endoscopic biopsies in patients with confirmed GVHD. By correlating symptoms with histopathologic findings, we aim to provide evidence-based guidance for tailoring endoscopic evaluation to individual patient presentations. This approach has the potential to optimize diagnostic accuracy, enhance patient care, and promote cost-effective use of health care resources.

## METHODS

### Study design and population

This retrospective cohort study evaluated the correlation between upper and lower GI symptoms and corresponding endoscopic evaluations with histopathologic diagnosis in patients with GVHD. The cohort included all patients who underwent HSCT at Montefiore Medical Center between January 1, 2015, and January 1, 2023. We analyzed patients with confirmed GVHD who presented with upper and/or lower GI symptoms and underwent endoscopic biopsy to determine whether symptom location can reliably guide the choice of endoscopy for diagnosis.

### Data collection

Data were extracted from the Clinical Looking Glass (CLG) database and the Epic electronic medical record system. The CLG database identified 300 patients with GVHD during the study period, of whom 75 met inclusion criteria. Chart reviews provided detailed clinical and outcome data. Follow-up data were collected for up to 1 year after the initial admission. All data were deidentified before analysis.

### Inclusion and exclusion criteria

Inclusion criteria comprised adults (18 years or older) with prior HSCT, and an *ICD-10* diagnosis of GVHD (D89.813), who presented with GI symptoms warranting endoscopic evaluation, and had at least 1 clinical encounter at Montefiore Medical Center. Patients were excluded if symptoms were attributable to other causes (e.g., medication-induced injury, ischemia, infection), if they had non–GI-related encounters post-HSCT, or were younger than 18 years.

### Outcomes

The primary outcome was the proportion of patients with biopsy-proven GVHD at anatomical sites correlating with presenting GI symptoms. Secondary outcomes included the association between symptom location and endoscopic findings, the agreement between overall histologic GVHD diagnosis and site-specific biopsy results (assessed using Phi coefficient). In addition, a subgroup analysis compared the diagnostic yield and cost-effectiveness of flexible sigmoidoscopy with full colonoscopy by evaluating biopsy results from rectal and sigmoid sites compared with segmental colon biopsies. All results were adjusted for sociodemographic covariates.

### Statistical analysis

All available data were included in the analysis. Categorical and continuous variables, including demographic characteristics, were summarized as frequencies and percentages. Associations between presenting GI symptoms and the type of endoscopy performed were evaluated using proportion measures.

The association between GI symptoms and endoscopic biopsy positivity was assessed using χ^2^ tests, with significance set at *P* < 0.05. The strength of correlation between symptom location and endoscopic diagnosis was evaluated using the phi coefficient for dichotomous variables.

Logistic regression models adjusted for potential confounders, including age, sex, race, ethnicity, income, and insurance status, were used to further assess the association between GI symptoms and endoscopic procedure selection.

To investigate diagnostic correlation between flexible sigmoidoscopy and a diagnosis of GVHD, phi coefficient analysis compared biopsy positivity from rectosigmoid sites with segmental colon biopsies.

All statistical analyses were performed using Stata/BE 19.0. This software facilitated calculation of χ^2^ tests, phi coefficients, and logistic regression models to ensure robust and reproducible results.

The sample size was determined by data availability in the CLG database between January 1, 2015, and January 1, 2023.

## RESULTS

Seventy-five patients met inclusion criteria and were analyzed. The mean age was 47.3 years, with a slight female predominance (56% female, 44% male). The cohort was ethnically diverse, reflecting the demographics of the Bronx, New York. Only 12% did not have insurance (Table 1). The mean interval between HSCT and GI GVHD diagnosis was 203 days, indicating variability in the timing of GI involvement.

**Table 1.** Baseline characteristics

Age	47.3 (mean)	SD: 16.98
Sex	N	%
Male	33	44
Female	42	56
Preferred language		
English	55	73.33
Spanish	19	25.33
Other	1	1.33
Race		
African American	23	30.67
White	21	28
Other	31	41.33
Ethnicity		
Caucasian	15	20
African American	22	29.33
Hispanic	34	45.33
Asian	0	0.00
Other	4	5.33
Employment status		
Employed	26	34.67
Unemployed	27	36
Other	22	29.33
Insurance status		
Private	22	29.33
Medicare	17	22.67
Medicaid	27	36
No insurance	9	12

Of the total cohort, 27 patients (36%) underwent both esophagogastroduodenoscopy (EGD) and colonoscopy as part of their diagnostic evaluation (Tables 2 and 4). Among these, 15 patients (55.6%) presented with symptoms affecting both the upper GI (UGI) and lower GI (LGI) tracts; 8 patients (29.6%) had symptoms confined to the LGI tract; and 4 patients (14.8%) had symptoms isolated to the UGI tract. Across the entire study population, 23 patients reported LGI symptoms, and 19 patients reported UGI symptoms.

**Table 2.** Symptoms and procedures performed

Symptoms	Procedure			Total
	EGD + colonoscopy	Colonoscopy only	EGD only	
	Total	Total	Total	
Both upper and lower GI	15 (93.75%)	1 (6.25%)	0 (0%)	16 (21.3%)
Lower GI only	8 (19.5%)	30 (73.1%)	3 (7.4%)	41 (54.7%)
Upper GI only	4 (22.22%)	0 (0%)	14 (77.78%)	18 (24%)

GI, gastrointestinal.

Biopsy results demonstrated strong anatomic correlation with presenting symptoms. Among patients with both UGI and LGI symptoms, 14 of 15 (93.3%) had positive histopathologic evidence of GVHD in biopsies from both regions. All 23 patients (100%) presenting with LGI symptoms alone had biopsy-confirmed GVHD localized to the LGI tract. Similarly, 17 of 19 patients (89.5%) with isolated UGI symptoms had positive biopsies from the upper GI tract. Only 1 patient (6.7%) with combined UGI and LGI symptoms showed biopsy positivity restricted to the LGI tract without upper GI involvement. Notably, 2 patients with UGI symptoms had negative upper GI biopsies, indicating that biopsy findings do not always correlate perfectly with symptoms (Table 3).

**Table 3.** Symptoms and biopsies performed in subgroups of patients having both EGD and colonoscopy performed (n = 27)

	N	%
Symptoms		
Both upper and lower GI	15	55.55
Lower GI only	8	29.63
Upper GI only	4	14.81
Biopsy		
Both upper and lower GI	26	96.30
Lower GI only	1	3.70
Upper GI only	0	0.00

GI, gastrointestinal.

Interestingly, 7 of 8 patients (87.5%) presenting with only LGI symptoms demonstrated histologic evidence of GVHD in both the LGI and UGI tracts, suggesting a possible subclinical or asymptomatic upper GI involvement clinical course in this group. Conversely, none of the patients with isolated UGI symptoms had positive biopsies from the lower GI tract, underscoring a directional relationship between symptoms and disease distribution (Table 3).

Endoscopic procedural choice was closely guided by symptoms on presentation. Among patients with both UGI and LGI symptoms, 15 of 16 (93.8%) underwent both EGD and colonoscopy. Among patients with LGI symptoms, 38 of 41 (92.6%) underwent colonoscopy, while all 18 patients with isolated UGI symptoms underwent EGD (Table 4).

**Table 4.** Symptoms and procedures performed in total cohort

	N	%
Symptoms		
Both upper and lower GI	16	21.34
Lower GI only	41	54.66
Upper GI only	18	24
Procedures		
EGD + colonoscopy	27	36.00
Colonoscopy only	31	41.33
EGD only	17	22.67
Biopsy		
Both upper and lower GI	26	34.67
Lower GI only	32	42.67
Upper GI only	17	22.67

GI, gastrointestinal.

Statistical analyses confirmed strong associations between symptoms and biopsy positivity. χ^2^ testing revealed significant relationships between UGI symptoms and positive EGD biopsies (*P* < 0.001) and between LGI symptoms and positive colonoscopy biopsies (*P* < 0.001). Phi coefficients further assessed the correlation between these variables, with *r* = 0.79 for UGI symptoms correlating with UGI biopsy positivity and *r* = 0.82 for LGI symptoms correlating with LGI biopsy positivity (both *P* < 0.001), indicating strong correlations.

Logistic regression models adjusting for demographic and socioeconomic factors, including age, sex, race, ethnicity, insurance status, and employment confirmed that symptom location remained a significant predictor of biopsy positivity (*P* < 0.001), supporting symptom-driven procedural decision making across diverse patient populations.

Finally, a subgroup analysis of 37 patients who underwent full colonoscopy for LGI symptoms compared biopsy results from rectosigmoid sites with those from the entire colon. Phi coefficient analysis demonstrated perfect correlation (*r* = 1.0, *P* < 0.001) between rectosigmoid and biopsy results with proven GVHD, suggesting that flexible sigmoidoscopy may be a cost-effective and less invasive alternative to full colonoscopy for diagnosing GVHD in patients with LGI symptoms (Table 5).

**Table 5.** Anatomic location of biopsies based on the type of endoscopic procedure performed

	N	%
EGD biopsy site-specific anatomic location		
Stomach	11	31.00
Stomach & duodenum	12	33
Duodenum	9	25
Esophagus	2	5.50
Esophagus and gastrointestinal tract	2	5.50
Colonoscopy biopsy site-specific anatomic location		
Rectum	23	41
Sigmoid	14	25
Rectum/sigmoid and remainder of the colon	14	25
Colon, excluding rectum and sigmoid	5	9

## DISCUSSION

In our cohort study evaluating the diagnostic yield of upper and lower endoscopy for GVHD, we found a strong association between presenting GI symptoms and the endoscopic approach used. The presence of UGI or LGI symptoms significantly influenced the selection of EGD, colonoscopy, or both. Importantly, symptom-guided endoscopic evaluation demonstrated high diagnostic accuracy, with strong correlations between symptom location and biopsy positivity.

χ^2^ analysis revealed significant associations between UGI symptoms and positive biopsy results via EGD (*P* < 0.001), as well as between LGI symptoms and positive findings on colonoscopy (*P* < 0.001). Phi coefficient analysis further underscored robust correlations: *r* = 0.79 for UGI symptoms with positive UGI biopsies and *r* = 0.82 for LGI symptoms with positive LGI biopsies (both *P* < 0.001). These associations remained significant after adjusting for confounding factors, including age, sex, race, ethnicity, insurance status, and employment, indicating that symptom-based procedural selection is effective across diverse patient populations.

**Figure 1.**
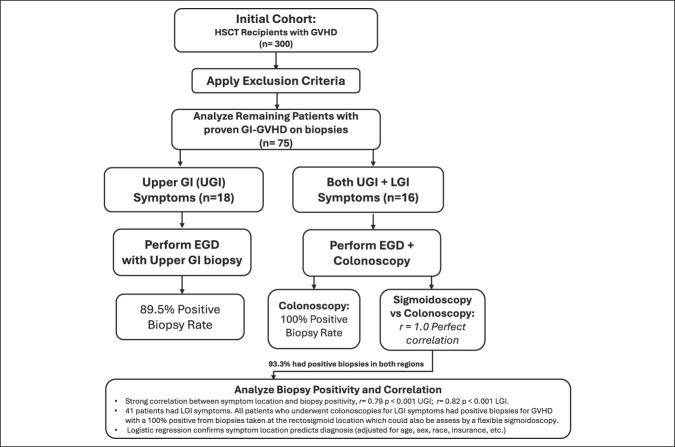
Protocol of our study and correlation between gastrointestinal symptoms and endoscopic biopsy results.

Anatomic correlation analysis demonstrated that rectal and/or sigmoid biopsies alone reliably diagnose GVHD in all patients undergoing full colonoscopy (*r* = 1.0, *P* < 0.001). This finding is both clinically and financially important, suggesting that flexible sigmoidoscopy with targeted rectosigmoid biopsy may be a cost-effective alternative to full colonoscopy for evaluating LGI symptoms in GVHD. Reducing the need for full colonoscopy could minimize procedural risks, decrease anesthesia exposure, shorten procedure times, and reduce health care costs without compromising diagnostic yield (6,7) (Figure 1).

Our results align with prior studies highlighting the patchy and multifocal nature of GVHD involvement in the GI tract and support a symptom-directed, anatomically targeted biopsy approach (8,9).

Despite the strengths of our multiculturally diverse cohort and comprehensive analyses, several limitations warrant consideration. First, the retrospective design and reliance on existing electronic medical records may introduce documentation biases. Second, the relatively small sample size (n = 75) limits statistical power and generalizability. Finally, as a single-center study, institutional practices and patient demographics may limit external validity.

In summary, our study reinforces the importance of anatomic correlation in the endoscopic diagnosis of GI GVHD and further suggests that a flexible sigmoidoscopy-based strategy may enhance cost-effectiveness while maintaining diagnostic accuracy in patients with GVHD who have LGI symptoms. However, these findings require further generalizability with multicenter trials with larger samples. If proven, these findings have the potential to refine endoscopic approaches in GVHD and improve patient care by balancing diagnostic thoroughness with resource utilization.

## CONFLICTS OF INTEREST

**Guarantor of the article:** Carlos Figueredo, MD.

**Specific author contributions:** C.F.: Data collection, Data analysis, Manuscript writing, editing. M.F.: Data analysis. L.B.: Manuscript editing, conceptualizing.

**Financial support:** None to report.

**Potential competing interests:** None to report.

**Study Highlights**

**WHAT IS KNOWN**

✓ Gastrointestinal graft-versus-host disease (GI-GVHD) is a common and serious complication of hematopoietic stem cell transplantation, and endoscopic biopsy remains the diagnostic gold standard for confirming gastrointestinal involvement.
✓ Existing literature provides conflicting evidence on whether presenting GI symptoms should guide endoscopic procedure selection, resulting in heterogeneous clinical practice and frequent use of combined upper and lower endoscopic evaluations.

**WHAT IS NEW HERE**

✓ Our study provides robust evidence that gastrointestinal symptom location strongly predicts site-specific biopsy positivity in GI-GVHD, demonstrating high anatomic correlation between upper GI symptoms and upper GI biopsies, and between lower GI symptoms and lower GI biopsies, independent of demographic and socioeconomic factors.
✓ Rectosigmoid biopsies alone showed perfect diagnostic concordance with full colonoscopy for lower GI GVHD, supporting flexible sigmoidoscopy as a reliable, less invasive alternative in patients with isolated lower GI symptoms.
✓ By leveraging a large, ethnically and socioeconomically diverse cohort, this study advances symptom-guided, anatomically targeted endoscopic strategies that may reduce procedural burden, anesthesia exposure, and healthcare costs without compromising diagnostic accuracy.

## ABBREVIATIONS:

GI: gastrointestinal
GVHD: graft vs host disease
HSCT: hematopoietic stem cell transplantation
LGI: lower gastrointestinal
UGI: upper gastrointestinal
